# Pollutant Dehalogenation Capability May Depend on the Trophic Evolutionary History of the Organism: PBDEs in Freshwater Food Webs

**DOI:** 10.1371/journal.pone.0041829

**Published:** 2012-07-27

**Authors:** Mireia Bartrons, Joan O. Grimalt, Guillermo de Mendoza, Jordi Catalan

**Affiliations:** 1 Center for Limnology, University of Wisconsin, Madison, Wisconsin, United States of America; 2 Institute of Environmental Assessment and Water Research, Consejo Superior de Investigaciones Científicas, Barcelona, Spain; 3 Centre for Advanced Studies of Blanes, Consejo Superior de Investigaciones Científicas, Blanes, Spain; 4 Centre de Recerca Ecològica i Aplicacions Forestals, Cerdanyola del Vallès, Spain; Ecole Normale Supérieure de Lyon, France

## Abstract

Organohalogen compounds are some of the most notorious persistent pollutants disturbing the Earth biosphere. Although human-made, these chemicals are not completely alien to living systems. A large number of natural organohalogens, part of the secondary metabolism, are involved in chemical trophic interactions. Surprisingly, the relationship between organisms’ trophic position and synthetic organohalogen biotransformation capability has not been investigated. We studied the case for polybromodiphenyl ethers (PBDE), a group of flame-retardants of widespread use in the recent years, in aquatic food webs from remote mountain lakes. These relatively simple ecosystems only receive pollution by atmospheric transport. A large predominance of the PBDE congener currently in use in Europe, BDE-209, largely dominated the PBDE composition of the basal resources of the food web. In contrast, primary consumers (herbivores and detritivores) showed a low proportion of BDE-209, and dominance of several less brominated congeners (e.g. BDE-100, BDE47). Secondary consumers (predators) showed large biomagnification of BDE-209 compare to other congeners. Finally, top predator fish characterized by low total PBDE concentrations. Examination of the bromine stable isotopic composition indicates that primary consumers showed higher PBDE biotransformation capability than secondary consumers. We suggest that the evolutionary response of primary consumers to feeding deterrents would have pre-adapted them for PBDE biotransformation. The observed few exceptions, some insect taxa, can be interpreted in the light of the trophic history of the evolutionary lineage of the organisms. Bromine isotopic composition in fish indicates that low PBDE values are due to not only biotransformation but also to some other process likely related to transport. Our finding illustrates that organohalogen compounds may strongly disturb ecosystems even at low concentrations, since the species lacking or having scarce biotransformation capability may be selectively more exposed to these halogenated hydrophobic semi-volatile organic pollutants due to their high bioaccumulation potential.

## Introduction

Prosperity in our civilization is partly supported by the continuous discovery of new chemicals. Some of them were developed with intentional toxicity (e.g., pesticides) but for others this quality is an undesired by-product. Whatever the original purpose, the persistence of toxic compounds in the environment may become a large-scale problem and the ecological and evolutionary consequences for the biosphere are largely unknown [1]. Insights into these consequences require a better understanding of the ecological and evolutionary constraints that modulate the accumulation, transformation and eventual fate in food webs of potentially toxic compounds.

A large proportion of persistent pollutants are synthetic organohalogen compounds. Although these molecules are new in nature, they are not completely alien to life. In fact, many marine organisms express biotransformation capabilities that utilize components of seawater, such as sulfate and halogens, to produce a variety of chemicals that may be useful either as consumption deterrents or as allelochemicals against other species [2]. There are more than 4,000 organohalogen compounds known to occur naturally and more than half of them contain bromine [3]. These secondary metabolites have been isolated from many organisms [4], particularly from algae [4], [5], [6], [7], [8], [9], sponges [10], marine worms [11], [12], [13], [14] and sea hares [15], [16]. The topic has been less studied in freshwater systems, although haloperoxidases have also been described in green algae (i.e., *Cladophora glomerata*) [17]. One may ask to what extent this previous ecological and evolutionary history related to natural organohalogen compounds may condition the response to current synthetic organohalogens. Here we present a comparative study on aquatic food webs in which we investigate the patterns of polybromodiphenyl ethers (PBDEs) across organisms of different trophic position and evolutionary history with the aim of bringing some light to this question.

Polybromodiphenyl ethers (PBDEs) are widely used as flame retardants. Their transport, bioaccumulation, biotransformation and fate in nature are matters of major interest due to their potential toxicity [18]. BDE-209, the main component of the decabromodiphenyl ether mixture (deca-BDE) currently in use, has been found in remote areas but little is known about its behavior in food webs [19]. PBDEs have been produced commercially in the form of diphenyl ether mixtures with three degrees of bromination: nominally penta-, octa- and deca-bromodiphenyl ether (BDE). However, the production of the former two formulations has been banned in the European Union [20] and Japan and voluntary withdrawn from the U.S. market due to growing environmental and human health concerns, such as the potential for nervous system damage, endocrine disruption and cancer [18], and the increasing concentrations of these compounds everywhere [19], [21], [22], [23], [24]. Deca-BDE mixture is mostly composed of BDE-209 (>97%), a molecule of extremely low volatility and, as a consequence, of expected scarce or null long-range atmospheric transport [25]. However, there is current evidence showing transport to remote areas [26], [27], [28], [29], [30]. Consequently, understanding PBDE bioaccumulation in food webs and its eventual fate is attracting an increasing amount of attention, particularly because the amounts produced are overtaking those of polychlorinated biphenyls (PCBs). PCBs are a paradigm of a global pollution legacy by persistent artificial substances [31]; they are distributed worldwide [32] despite being banned in the 1970 s, and still bioaccumulate in food webs [33].

We studied the patterns of PBDE distribution throughout the food webs of four high mountain lakes in the Pyrenees (Spain). Remoteness and altitudinal isolation of these lakes assure exclusive PBDE pollution via atmospheric transport. Relatively simple food webs allowed for a comprehensive approach from basal resources to top predators (Table 1). Our findings reveal an enhanced biotransformation capability of brominated flame-retardants (PBDE) in primary consumers compared to higher trophic levels. Given the short period for which these compounds exist in nature, this suggests a case of pre-adaptation built on previous evolutionary history related to chemical ecological interactions.

**Table 1:** Organisms and assemblages analyzed in this study.

Organism or assemblage	Lineage	Trophic level	Lake
*Nostoc*	Cyanobacteria	BR (only PP)	C, V
Epilithon (mainly diatoms and cyanobacteria)	–	BR (mostly PP)	Le, Lo, C, V
Epipelon (diatoms, cyanobacteria and heterotrophic bacteria)	–	BR (PP+D)	Le, Lo, C, V
Top sediment (bacterial biofilm with some microalgae)	–	BR (mostly D)	Le, Lo, C, V
Nematoda	Nematoda	SC (mostly)	Le
Oligochaeta (bottom)	Annelida, Oligochaeta	PC	Le, Lo, V
Oligochaeta (littoral)	Annelida, Oligochaeta	PC	Le, Lo, C, V
*Pisidium* (bottom)	Mollusca, Bivalvia, Veneroida	PC	Lo, V
*Pisidium* (littoral)	Mollusca, Bivalvia, Veneroida	PC	Le, Lo, C
*Ancylus fluviatilis* Müller, 1774	Mollusca, Gastropoda, Pulmonata	PC	Lo, C
*Radix peregra* (Müller, 1774)	Mollusca, Gastropoda, Pulmonata	PC	Lo, C, V
Planktonic crustaceans (*Daphnia longispina* (Müller, 1785), *Eudiaptomus vulgaris* (Schmeil, 1896), *Cyclops abyssorum* Sars, 1863)	*Arthropoda, Crustacea*	SC + PC	*Le, Lo, C, V*
*Aeschna*	Insecta, Odonota, Anisoptera	SC	Le
Zygoptera	Insecta, Odonota, Zygoptera	SC	Le
*Sialis lutaria* (Linnaeus, 1758)	Insecta, Megaloptera	SC	Le, Lo, C, V
Limnephilidae (*Annitella, Potamophylax, Limnephilus)*	Insecta, Trichoptera, Integripalpia	PC	Le, Lo, C, V
*Mystacides azurea* (Linnaeus, 1761)	Insecta, Trichoptera, Integripalpia	PC	Lo
*Polycentropus flavomaculatus* (Pictet, 1834)	Insecta, Trichoptera, Annulipalpia	SC	Le, Lo, C, V
*Boreonectes* (adult) (formerly *Stictotarsus*)	Insecta, Coleoptera, Dytiscidae	SC	V
*Haliplus* (adult)	Insecta, Coleoptera, Haliplidae	PC	V
Chironomidae (other than Tanypodinae) (bottom)	Insecta, Diptera, Chironomidae	PC	Le, Lo, V
Chironomidae (other than Tanypodinae) (littoral)	Insecta, Diptera, Chironomidae	PC	Le, C, V
Tanypodinae	Insecta, Diptera, Chironomidae	SC	Le, Lo, C, V
Ceratopogonidae	Insecta, Diptera, Ceratopogonidae	SC	Le
Hydracarina	Arachnida	SC	V
*Phoxinus* sp.	Chordata, Actinopterygii, Cypriniformes,	SC	Le, Lo
*Salmo trutta* Linnaeus, 1758	Chordata, Actinopterygii, Salmoniformes	SC	Le, Lo, C, V

The evolutionary lineage and trophic information is provided. Primary consumers (PC) include herbivores and detritivores feeding on basal resources (BR), which in these lakes are mostly biofilms of varying degree of autotrophic primary producers (PP) and heterotrophic decomposers (D). Secondary consumers (SC) include organisms predating upon primary or other secondary consumers. In a few cases, we have distinguished between assemblages in different parts of the lake (e.g., littoral or bottom) or contrasting microhabitats (e.g., epilithon, epipelon). Lake occurrence is indicated by: Le, Llebreta; Lo, Llong, C, Colomina and V, Vidal.

## Materials and Methods

### Study Site and Sampling

Samples were collected in four high mountain lakes from the Pyrenees (Catalonia, Spain): Llebreta (42.55°N 0.89°E, 1620 m a.s.l.), Llong (42.57°N 0.95°E, 2000 m), Xic de Colomina (42.52°N 0.99°E, 2425 m) and Vidal d’Amunt (42.53°N 0.99°E, 2688 m). They are softwater oligotrophic lakes, with long ice cover periods (from ca. 4 to 7 months) and cold-water temperature during ice-free periods. These conditions involve relatively simple food webs, scarce biomass and relatively slow development of aquatic insects.

The lakes are located in the Aigüestortes i Estany de Sant Maurici National Park, all necessary permits were obtained for the described field studies. Sampling was performed in July 2004. We attempted to cover all main trophic compartments and organisms: from basal resources to fish (Table 1). Basal resources included some macroscopic algae (i.e., *Nostoc*), epilithon (rock biofilms with diatoms, cyanobacteria and green algae), epipelon (biofilms on littoral sediments similar in composition to rock biofilms but with a higher proportion of heterotrophic prokaryotes) and top sediment (deep bacterial biofilms with some microalgae). Phytoplankton could not be measured due to the difficulty for obtaining enough material in these highly oligotrophic lakes. However, we were able to collect sufficient amount of zooplankton for analysis. All sort of habitats were examined for macroinvertebrates. Brown trout (*Salmo trutta*) was sampled using gill nets and results were already included in a previous publication [34].

Epilithon was scraped from ten stones distributed throughout the whole littoral shore of each lake. A metallic ultra–cleaned brush was used. Large colonies of the cyanobacteria *Nostoc* were collected manually with metal tweezers. Surface sediment was sampled with a plastic cylindrical tube in the littoral depths (epilithon) and a Kajak gravity core in the deep parts (top sediment). Top layers of 1–2 cm thickness were sliced from the sediment cores. Zooplankton was sampled by vertical tows using a 160 µm mesh zooplankton net near the maximum depth point. The littoral macroinvertebrates were collected in each lake by extensive kick sampling throughout the perimeter during several hours. Macroinvertebrates from the maximum depths of the lakes were collected using an Ekman sampler. Samples were kept frozen (−20°C) until examination. The taxonomic resolution in which the results are reported has been a compromise between achieving as much taxonomic detail as possible and having enough material for PBDE composition analysis.

### Chemicals and Standards

N-Hexane, dichloromethane, isooctane, concentrated sulfuric acid and acetone were purchased from Merck (Darmstadt, Germany). PCB#142, PCB#200 and PCB#209 were purchased from Dr. Ehrenstoffer (Augsburg, Germany). Standards of fourteen polybrominated diphenyl ethers (BDE-17, BDE-28, BDE-47, BDE-66, BDE-71, BDE-85, BDE-99, BDE-100, BDE-138, BDE-153, BDE-154, BDE-183, BDE-190 and BDE-209) in isooctane were purchased from Cambridge Isotope Laboratories (Andover, MA, USA). There are no standards for BDE-35, BDE-77, and BDE-156. The peaks of these compounds were identified using relative retention time predicted from a model developed to estimate retention times of the 209 individual PBDE congeners [35] and the mass spectrum. Their behavior in gas chromatography was assumed similar to those chemically closer for which we had standards.

### PBDE Analysis

The extraction and clean-up procedure is described in detail elsewhere [36]. Samples were spiked with PCB#209 standards and extracted by sonication with hexane-dichloroethane (4∶1) in four successive steps of 15 min. Clean-up was performed by sulfuric acid oxidation (4 times). The solutions were concentrated to near dryness under a gentle flow of nitrogen and redissolved in 50 µl of isooctane. Before chromatographic analysis, internal standards of PCB#142 and PCB#200 were added.

Samples were analyzed by gas chromatography coupled to negative ion chemical ionization mass spectrometry (GC-NICI-MS) in a TRACE GC ULTRA (Thermo Electron, Milan, Italy) coupled to a MS DSQ (Thermo Electron, Austin, Texas, USA) [37]. The system was equipped with a DB-5MS capillary column (15 m×0.25 mm×0.1 µm film thickness) coated with phenyl arylene polymer that is virtually equivalent to 5% phenyl 95% methylpolysiloxane stationary phase. The oven temperature program was from 140°C (held for 1 min) to 325°C at 10°C·min^−1^ (held for 10 min). Helium was used as a carrier gas (1 mL·min^−1^) and ammonia as an ionization gas (2.4·10^−4^ Pa). Transfer line temperature was 300°C. Quantification was performed from the intensities of the m/z 79 ion [Br]^−^. Confirmation ions were m/z 81 [Br]^−^, 161 [HBr_2_]^−^ and 327, 405, 483, 563 and 643, corresponding to either [M]^−^ or [M-HBr_2_]^−^. BDE-209 was measured from the intensities of the m/z 487 ion [Br]^−^ and the confirmation ion was m/z 489 [Br]^−^.

### Bromine Stable Isotope Ratios

We use an innovative application of Br stable isotopes (Br^79^ and Br^81^) for evaluating biotransformation and constrained transport. Br^79^ and Br^81^ occur in similar amounts in nature [38] and PBDEs in industrial products and standards show also Br^79^:Br^81^ ratios close to 1 [39]. This means that, for instance, in the case of BDE-209, the most likely combination in a molecule is 5 and 5 atoms of each isotope. However, in the mixture there are molecules with all combinations 4∶6, 6∶4, 3∶7, 7∶3, …, with a decreasing probability, respectively. We hypothesized that, when enzymatic debromination occurs, the smaller the molecules, the easier will be attacked by enzymes. Therefore, the Br^79^:Br^81^ ratio will progressively decline in the remaining pool of the molecule (e.g. BDE-209) and will increase in the pool of the resulting less brominated molecules. Furthermore, the pools of the derived molecules will experience the same phenomenon. In summary, the larger the enzymatic biotransformation action over a certain BDE initial pool, the lower the Br^79^:Br^81^ ratio of the pool. Consequently, we could expect declining ratios throughout the food web if feeding is the main pathway of PBDE consumer bioaccumulation, as at each trophic level they will be processing a pool of substances that already have suffered discrimination in the previous trophic level. The more intense the enzymatic debromination at certain trophic level, the larger the decline of the Br^79^:Br^81^ ratio between this trophic level and the previous one. Other processes may also cause discrimination between PBDE molecules of different Br isotopic composition during transport within or between organisms. In particular, facilitated transport across membranes or slow diffusion may favor lighter molecules, thus increasing the Br^79^: Br^81^ ratio. We estimated the Br^79^: Br^81^ ratio for BDE-209 and BDE-47 pools, using the fragments m/z 79 and 81, respectively, in the NICI-MS spectra.

### Quality Assurance

Procedural blanks were analyzed for every set of six samples, which corresponded to periods between twelve hours and two days of sample handling, depending on the matrix. The recoveries of the surrogate standard, PCB-209, were calculated for each sample, being 69±22% (average ± standard deviation). The recovery of this surrogate was used for correction of the concentrations of PBDE congeners in each sample. Identification and quantification of PBDE congeners was performed by injection of external standards at different concentrations. Relative responses to PCB-200 were used in order to correct for instrumental variability and this value was corrected by the recovery of the surrogate standard (PCB-209). Limits of detection and quantification were calculated from real samples, as the mean of the noise signal plus 3 and 5 standard deviations, respectively. They ranged between 0.1–0.8 and 0.2–1.3 pg g^−1^, respectively. Final validation was made by analysis of reference material obtained from the Arctic Monitoring and Assessment Program (AMAP). We participate regularly in the AMAP Ring Test Proficiency Program for POPs (Centre de Toxicologie, Institut National de Santé Publique du Québec, Québec, Canada) and the laboratory results usually were within 20% of the consensus values, including BDE-209 concentrations.

### Numerical Methods

To compare the PBDE composition between trophic levels or samples grouped in different ways, because of the skewed distribution of the values, we applied PERMANOVA, a non-parametric method for multivariate analysis of variance using Bray-Curtis distance [40]. The analysis was performed in R version 2.14.1, using Vegan package. To test whether BDE-209 high (low) values distribute randomly between primary, and secondary consumers we used Fisher exact test performed in S-PLUS 6.1 package. To compare Br^79^: Br^81^ ratios between trophic levels we performed a One-way ANOVA followed by the Tukey’s method for multiple paired comparisons in S-PLUS 6.1 package. Finally, we performed principal component analysis (PCA) to ordinate the variability observed among basal resources and invertebrate consumers into a few main components. The analysis was performed using CANOCO 4.5 and biplots using CanoDraw 4.0.

## Results and Discussion

### Trophic Level and PBDE Content

**Figure 1:**
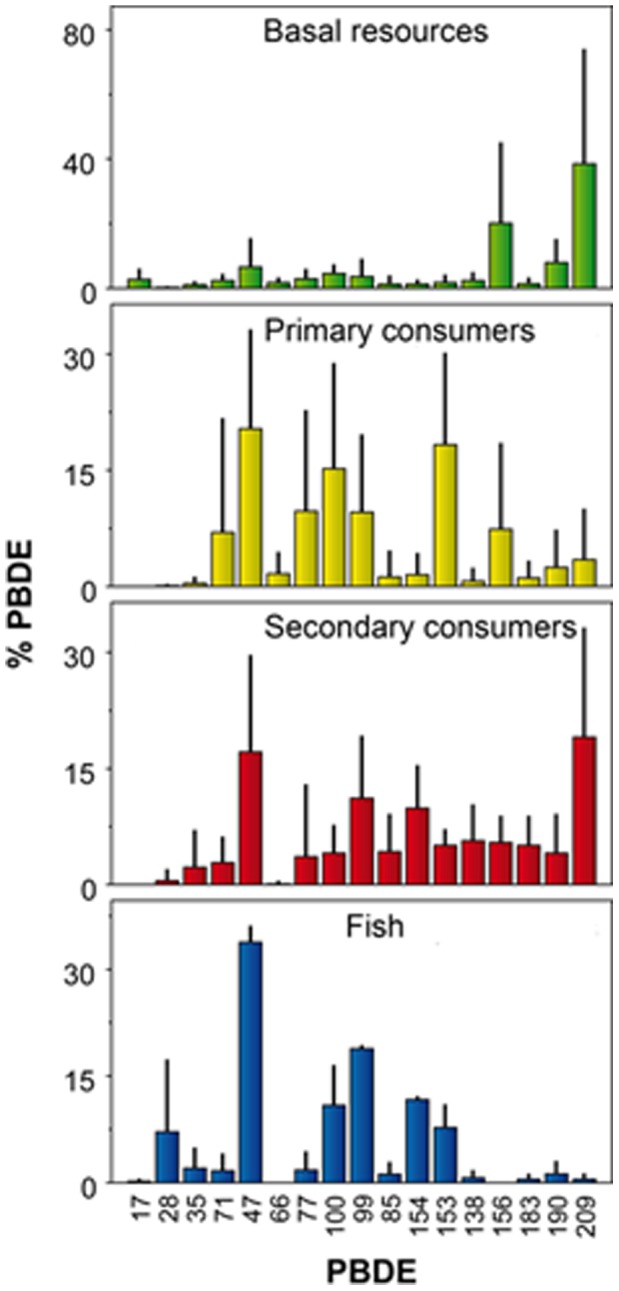
PBDE relative composition at different trophic levels of the lake food webs studied. Bars indicate average percentages of each PBDE in the lake food web components included in the respective trophic category. Error bars indicate standard deviations. Different bar colors are used to identify the trophic levels across figures. BDE-209 is the current congener produced industrially in Europe. Congeners BDE-35, BDE-77 and BDE-156 have never been present in any industrial mixture.

**Figure 2:**
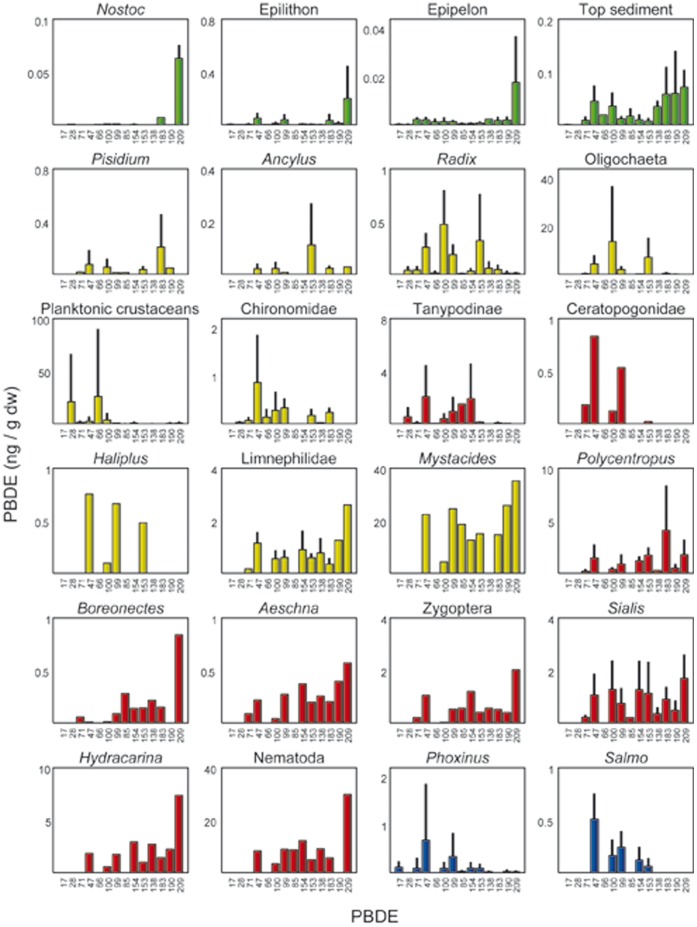
PBDE content in individual taxa and assemblages of the lake food webs studied. Mean and standard deviation are indicated, if more than one sample was available. The complete list of measured values is provided as Table S1. Bar colors correspond to basal resources (green), primary consumers (yellow), secondary consumers (red), and fish (blue). Error bars correspond to standard deviation of the whole number of samples analyzed for a given species or assemblage.

PBDEs were found at all the trophic levels analyzed, from basal resources to secondary consumers and fish (Table 2). Total PBDE average concentration differ between trophic levels (basal resources, 0.2 ng g^−1^dw; primary consumers, 18; secondary consumers, 12; and fish 2.3, respectively). However, mean values are largely influenced by the skewed distribution of concentrations (Table 2). The general bioaccumulation pattern is better illustrated by the median of total PBDEs (basal resources, 0.08 ng g^−1^dw; primary consumers, 1.01; secondary consumers, 2.84; and fish, 0.59, respectively) and of individual congeners (Table 2). The increase is about one order of magnitude between basal resources and primary consumers, and nearly triplicates between the latter and secondary consumers. However, fish show lower concentrations that their food items indicating that other processes are involved. In fact, not all congeners show individually a characteristic bioaccumulation increase. The dominant congeners vary between trophic levels (Fig. 1) and a large standard deviation around the mean (Table 2) reflects the high variability among taxa of the same trophic level (Fig. 2). Particularly striking is the variability of PBE-209 between trophic levels. The PERMANOVA indicated significant differences among trophic levels in PBDE profiles (p<0.001), subsequent pair comparisons showed that basal resources composition differ from both primary (p<0.003) and secondary (p<0.017) consumers and that the two types of consumers differ significantly between them (p<0.011). In contrast, fish PBDE composition was not significantly different from any of the other three levels (basal resources, p<0.06; primary consumers, p<0.23; secondary consumers, p<0.14). The p-values reflect that fish PBDE composition was closer to primary consumers than to secondary ones. The PBDE profiles of the two fish species analyzed (*Salmo trutta*, *Phoxinus* sp.) were similar (p>>0.1) (Fig. 2).

**Table 2:** PBDE concentration at different trophic levels.

	Basal resources		Primary consumers		Secondary consumers		Fish	
ng g^−1^ dw	mean ± SD	median	mean ± SD	median	mean ± SD	median	mean ± SD	median
BDE-209	0.1±0.17	0.04	0.88±5.25	*bdl*	2.74±7	0.63	0.01±0.02	*bdl*
BDE-190	0.02±0.04	0.01	0.61±3.85	*bdl*	0.33±0.56	0.05	0.02±0.04	*bdl*
BDE-183	0.03±0.04	0.01	0.43±2.19	0.01	1.01±2.01	0.14	*bdl*	*bdl*
BDE-156	0±0.01	*bdl*	0.27±1.63	*bdl*	0.7±1.71	0.18	0.01±0.01	*bdl*
BDE-138	0.01±0.01	*bdl*	0.04±0.18	*bdl*	0.77±2.17	*bdl*	0.01±0.02	*bdl*
BDE-153	*bdl*	*bdl*	1.67±4.35	0.22	0.77±1.28	0.15	0.13±0.13	0.09
BDE-154	0±0.01	*bdl*	0.34±1.88	*bdl*	1.49±2.89	0.26	0.19±0.26	0.09
BDE-85	0±0.01	*bdl*	0.44±2.78	*bdl*	0.63±2.06	*bdl*	0.02±0.03	0.01
BDE-99	0.01±0.03	0.01	1.4±4.37	0.19	1.1±2.06	0.40	0.43±0.5	0.12
BDE-100	0.01±0.02	*bdl*	6.4±26.84	0.31	0.5±0.86	0.18	0.22±0.32	0.07
BDE-77	0.02±0.04	*bdl*	0.84±2.91	0.04	0.19±0.51	*bd*	0.06±0.11	*bdl*
BDE-66	*bdl*	*bdl*	0.27±1.44	*bdl*	0±0.01	*bdl*	*bdl*	*bdl*
BDE-47	0.03±0.04	0.01	1.53±3.77	0.24	1.41±2.03	0.76	0.86±1.14	0.20
BDE-71	0±0.01	*bdl*	2.81±16.83	*bdl*	0.11±0.12	0.09	0.06±0.17	*bdl*
BDE-35	*bdl*	*bdl*	0.08±0.33	*bdl*	0.16±0.33	*bdl*	0.08±0.21	0.01
BDE-28	*bdl*	*bdl*	0.01±0.03	*bdl*	0.06±0.24	*bdl*	0.14±0.37	*bdl*
BDE-17	*bdl*	*bdl*	*bdl*	*bdl*	*bdl*	*bdl*	0.02±0.06	*bdl*
Samples	18		55		18		23	

*bdl,* below detection limit.

Basal resources showed a large general dominance of BDE-209 (Fig. 1). This overwhelming dominance of BDE-209 in basal resources indicates that this congener is largely the main component of the PBDE atmospheric input at these sites, which is in agreement with the deca-BDE industrial mixture currently in use in Europe [20]. The occurrence of other congeners was mostly related to the degree of autotrophy of the resource (Fig. 2). That is, there were only traces of other congeners in the *Nostoc* colonies; other congeners were largely a minority in the epilithon and epipelon samples, and they were relatively abundant in top sediment biofilms (Fig. 2). Top sediment biofilms are enriched in less brominated congeners compare to the other biofilms, probably because bacterial dehalogenation of PBDEs requires anaerobic conditions [41], [42]. These high altitude lakes are exposed to high ultraviolet radiation (UV); however, UV cannot be responsible for the enrichment in less brominated congeners in top sediments. Top sediment biofilms were sampled from the deepest part of the lake; therefore, UV reaching these biofilms was much less than biofilm sampled at the littoral zone (i.e., epipelon and epilithon), in which BDE-209 is largely the dominant congener (Fig. 2). In a previous study, it was shown that there are no differences in PBDE composition between rock biofilms exposed to full radiation (extremely high UV) and biofilms from the same rocks located in the shaded parts, which receives only diffuse radiation [29].

The proportion of less brominated congeners increased markedly in all consumers compared to basal resources (Fig. 1). However, the lower levels and proportion of BDE-209 in primary consumers contrast with relatively high proportion in secondary consumers (Table 2, Fig. 1). A Fisher’s exact test indicated that the distribution of high values of BDE-209 between species of the two consumer types was not random (p<0.15). No habitat effect can be attributed to these patterns. Primary consumers living both in littoral, and deep sediment habitats (i.e., *Pisidium*, Oligochaeta, Chironomidae) showed similar PBDE profiles for both populations (see Table S1), no significant differences existed in paired comparisons (p>>0.5). In addition, secondary consumers from a given habitat (e.g., littoral) showed contrasting patterns with their potential preys (p<0.006).

### PBDE Biotransformation Capability

The pattern found in consumers is not the one we should expect, assuming simple bioaccumulation from initial trophic sources. The increase in less brominated congeners and decline in BDE-209 suggest that debromination capability is common among many food web components. The presence of some congeners historically absent from industrial mixtures [39] supports this interpretation (i.e., BDE-35, BDE-77 and BDE-156). Very low levels of BDE-209 in primary consumers indicate that PBDE biotransformation is enhanced at this trophic level (Fig. 1).

**Figure 3:**
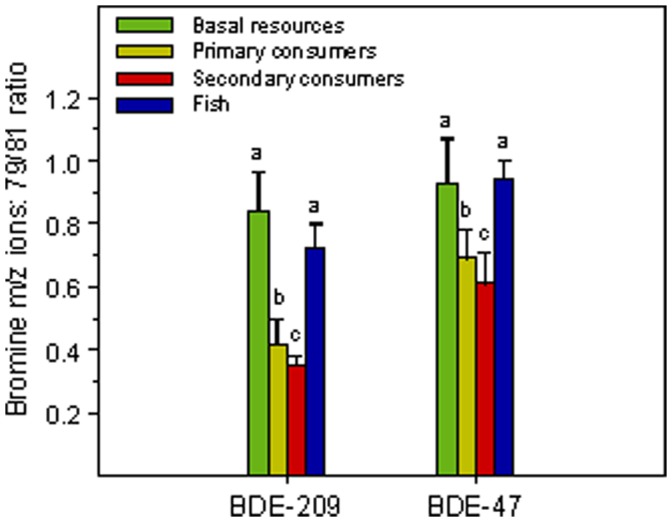
PBDE isotopic bromine ratios (Br^79^:Br^81^) across trophic levels. The ratio Br^79^:Br^81^ in nature and industrial PBDE mixtures is close to one. Deviations from that ratio can be used as indication of processes determining isotopic fractionation. Mean and standard deviation for two BDE congeners are plotted for each trophic level: BDE-209, for being the initial industrial source; and BDE-47, because is the resulting most abundant congener in the food web. The ratio differences between trophic levels for both compounds are significantly different as a whole (p<0.001); paired comparisons are indicated in the figure using lower case letters, only basal resources and fish ratios do not differ for a 95% interval of confidence. Higher Br^79^:Br^81^ values in BDE-47 than in BDE-209 and declining values from basal resources to secondary consumers are compatible with the existence of accumulative effects of enzymatic debromination. High Br^79^:Br^81^ values in fish indicate that some other additional process is taking place in them that discriminates against the heavier isotope, differential transport is suggested as a potential mechanisms, either at gut uptake or during within body distribution.

**Figure 4:**
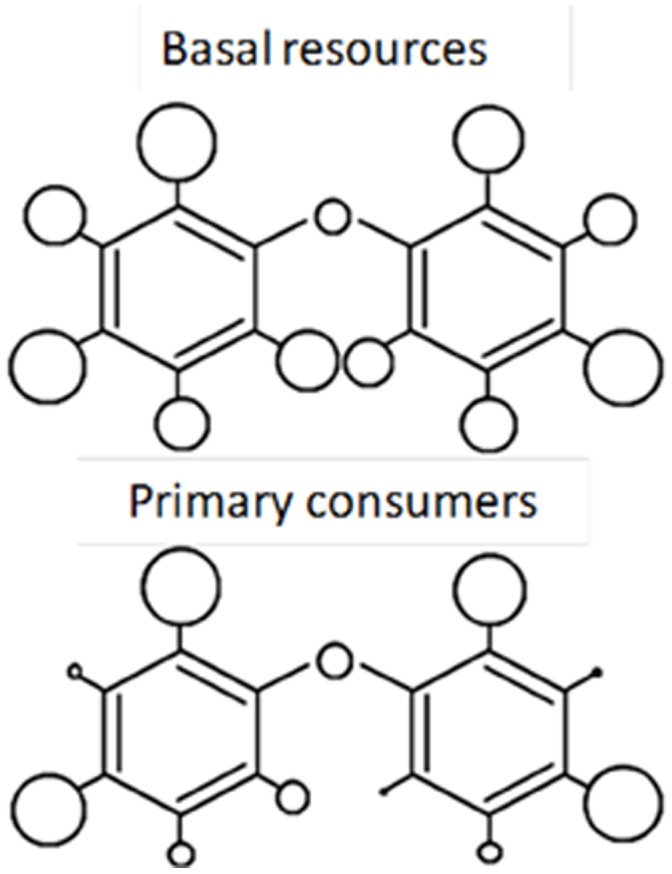
Frequency of bromine atoms at each potential site of the BDE molecule. The larger the circle, the higher the frequency. The frequency at which a potential site in the BDE molecule was occupied by a bromine atom was estimated from the formula of the respective PBDE congeners and their proportion in a trophic level. Basal resources and primary consumers of the lake food web studied are compared. Interestingly, the molecule sites with higher occupancy correspond to those that are chemically more stable. The result is compatible with a high enzymatic biotransformation capability in primary consumers.

Bromine isotopic composition of PBDEs provides further evidence that progressive dehalogenation occurs within the food web, starting from BDE-209 (Fig. 3). Higher proportion of the lighter isotope in BDE-47 than in BDE-209 at each trophic level agrees with the interpretation that BDE-47 results from the progressive biotransformation of the originally fully brominated molecule (Fig. 3). Br^79^:Br^81^ ratio decreases in the BDE source pool and, correspondingly, increases in the pool of the debrominated form. On the other hand, for both BDE-29 and BDE-47, the ratio decreases with increasing trophic level (Fig. 3), indicating that at each higher level the PBDE pool had a longer biotransformation trajectory. Therefore, the two isotopic features support that the patterns observed along the food web are highly influenced by the biotransformation capability of the organisms. If this were so, the frequency of BDE congeners would reveal which bromine sites in the BDE molecule are more liable to biotransformation (Fig. 4). For primary consumers, bromines in the 2 (ortho) and 4 (para) positions (e.g., penta BDE-99 and tetra BDE-47) are most frequent, which in fact correspond to the positions that are chemically more stable [43].

Interestingly, the ratio found in BDE-209 of microbial biofilms is close to one (Fig. 3). As mentioned in material and methods, PBDEs in industrial products and standards show ratios between the two bromine stable isotopes (Br^79^ and Br^81^) close to this value. This indicates that BDE-209 photodegradation during atmospheric transport does not result in isotopic fractionation. The atomic weight differences of the two isotopes are relatively small (compared for instance to the stable isotopes of lighter elements, e.g. C, N). This slight difference does not seem to produce any significant fractionation during photodegradation according to our results. However, the isotopic size difference, and most probably the aggregate difference in a molecule rich in one of the isotopes, does result in fractionation under enzymatic attack.

The low BDE concentrations in both fish species (*Phoxinus* and *Salmo*) compared to other organisms with much shorter life span, and the higher PBDE compositional resemblance of fish with primary consumers than with secondary ones, indicate that fish merit further consideration. There are several causes that can explain lower PBDE concentrations in fish, which are not mutually exclusive: a larger biotransformation capability; a limited gut uptake; and/or a preferential accumulation in some organ. Fish show a completely different Br^79^:Br^81^ ratio than their prey and any other animal in the food web, which contrasts with the scarce PBDE compositional differentiation. The Br^79^:Br^81^ ratios are much higher than in primary and secondary consumers (Fig. 3), close to the values found in basal resources. This indicates that there has been a strong fractionation in favor of the lighter isotope during the food pathway, if PBDE uptake is largely by feeding, which seems the most likely way according to the properties of these compounds. The high fractionation may happen if BDE transport in the fish gut is highly limited compared to invertebrates, which we suggest as a hypothesis for further investigation. This will also explain a lower concentration. However, at the light of our results, we cannot discard accumulation in some other organ (e.g., liver as found for rats [44]), as we only measured muscle; in that case, the isotopic fractionation may happen during internal transport. In rats, the BDE-209 absorption efficiency has been estimated as 20% [44], whether or not fish has a similar value, and whether this is sufficient for such a high fractionation, requires further investigation. The high Br^79^:Br^81^ ratio of BDE-47 in fish is compatible with both a high transport fractionation of the congener or high rates of dehalogenation from BDE-209 and other highly brominated congeners that suffered the fractionation.

Biological debromination reactions are still poorly understood. At present, there are different non-exclusive hypotheses based on cytochrome P450 (involved in the metabolism of lipophilic xenobiotics and plant allelochemicals) [45], iodothyronine deiodinase [46] (involved in the regulation of thyroid hormones) or endobiont bacteria [47], as the main implicates. Debromination capability has been reported in bacteria [48], [49] and chordates such as fish [46] and some mammals (rats [44], [50], [51], bottlenose dolphins (*Tursiops truncatus*) [52], beluga whales (*Delphinapterus leucas*) [51], polar bears (*Ursus maritimus*) [53], [54] and lactating cows [55]). Our results show that PBDE transformation also occurs in many groups of invertebrates phylogenetically distant (i.e., mollusks, oligochaetes, crustaceans, insects and water mites). A first important conclusion from this study is that PBDE may be much less persistent in nature than up to here assumed.

### Trophic Evolutionary History

Our results show that high PBDE biotransformation capability is not universal among organisms. It appears preferentially related to primary consumers, yet not exclusively. Since it is unlikely that PBDE biotransformation capability evolved in so many different groups as a response to the recent anthropogenic release of brominated organic compounds into the environment, we must conclude that showing (or lacking) the capability is related to the previous eco-evolutionary history of each organism.

The high capability for biotransforming PBDEs shown by some organisms may be rooted in the structural similarity of these compounds to natural bromohalogens [4], which are used as feeding deterrents by some primary producers and microbes within aquatic food webs [56]. We suggest that high PBDE debromination capability in primary consumers can be seen as a preadaptation case [57]. Response to a previous selection pressure (halogenated feeding deterrents) would have unexpectedly prepared them for biotransformation of human-made toxicants (PBDEs). Correspondingly, secondary consumers would be less capable of debrominating PBDEs and, therefore, they are more susceptible to PBDE bioaccumulation, particularly of highly hydrophobic compounds such as BDE-209.

**Figure 5:**
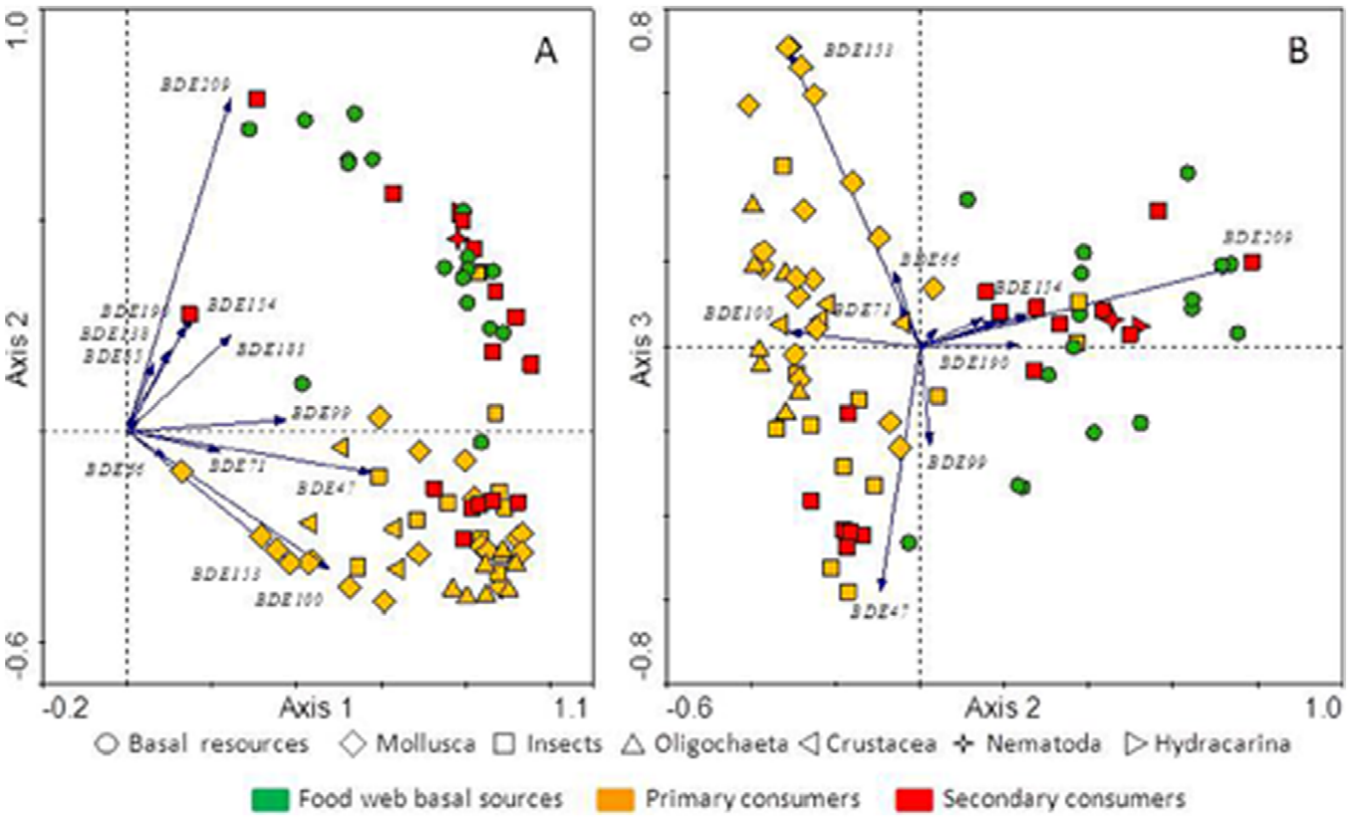
Ordination of the PBDE profiles in basal sources and invertebrates of the lake food webs studied. The variation of the PBDE composition was summarized by means of a principal component analysis (PCA) using the Hellinger distance [63]. Biplots of the first two principal components (A) and the second and third (B) are shown. Symbols refer to each sample analyzed and vary to show the corresponding trophic level (color) and taxonomic position (shape) as indicated in the legend. With a few exceptions primary and secondary consumers are discriminated between them.

An ordination of the PBDE values across all samples analyzed, excluding fish, evidenced further details of the differences among trophic levels (Fig. 5). BDE-47 and BDE-209 mostly define the main two axes of variation and highly discriminate between primary and secondary consumers, and show the degree of variation within each trophic level (Fig. 5A). The third axis of variation indicates that insects accumulate mainly BDE-47 and BDE-99; whereas mollusks, oligochaetes and crustaceans, show higher relative BDE-153 and BDE-100 content (Fig. 5B). This may reflect different debromination enzymatic mechanisms. Interestingly, crustaceans and insects do not show the same congener profile, which suggests that the latter may have evolved the capability de *novo*, at least partially. This may be important to understand variability within insects, as we will comment below.

In the case of planktonic crustaceans, our samples were a mixture of secondary consumers (*Cyclops*, predator) and primary consumers (*Eudiaptomus* and *Daphnia*, grazers), with the former predominating in biomass. BDE-209 was low comparing to other congeners (see Table S1), which were very high (Fig. 2). Because of the mixture of primary and secondary consumers in the samples, no definitive judgment can be made about these results. However, as predator copepods were the most abundant, we may tentatively assume that debromination capability is also present in the copepod predators. Copepods as a group have an epibenthic herbivore origin [58] (Cambrian) and it may happen that all genera (including predators such as *Cyclops*) might possess debromination capability.

Insects analyzed mostly followed the general patterns expected from their trophic position, but the ordination also indicated that some of them showed PBDE profiles different to that expected from the overall pattern (Fig. 5). Among secondary consumers, ceratopogonids and tanypodine chironomids showed PBDE profiles closer to primary consumers; whereas among primary consumers some Trichoptera (Limnephilidae and *Mystacides*) showed secondary consumer patterns. In contrast to crustaceans, there is not an evolutionary history in insects linking marine ancestors directly to freshwater aquatic forms. There was an early transition of predatory organisms from sea to land and the insect freshwater life histories of immature forms evolved later [59]. Therefore, the ecological trophic role of bromine compounds in aquatic food webs was probably largely interrupted and the chances of disappearance of debromination capability increased, if it was ever present in the ancestor. Many aquatic insects are still predators and, therefore, they have not been exposed to brominated feeding deterrents, which could explain their low (null?) debromination capability (Fig. 2, 5). Of the two coleopterans analyzed, each showed the respectively expected PBDE pattern: *Haliplus* as primary consumer and *Boreonectes* as a predator. This fact indicates that, at least in some cases, there has been time enough to evolve debromination capability. Indeed, Haliplidae and Dytiscidae common relative diverged at early Triassic. Therefore, we hypothesize that the exceptions we find between trophic mode and debromination capability correspond to insect taxa that have evolved relatively recently from organisms with a different feeding mode.

Do exceptions correspond to taxa with evolutionarily recent trophic shifts? Among the primary consumers analyzed, only two trichopteran taxa did not show biotransformation capability. The species involved are mainly shredders of plant debris and belong to the families Limnephilidae and Leptoceridae (*Mystacides*), which are among the ones with the broadest ecological distribution within the order. Only about 8% of the trichopteran families are purely predators; however, they correspond to the oldest evolutionary families [60]. Predation was probably the feeding mode of the ancestor lineage of the order [59]. Currently, periphyton scrapers comprise about 25% of the families in Trichoptera, yet most of them are evolutionarily recent groups [60]. Therefore, the bulk of the trichopteran families, which are shredders of plant debris or omnivorous, probably has not had a long enough evolutionary history that included interactions with aquatic organisms producing brominated compounds. Furthermore, many of them are consumers of debris rather than fresh material, thus no real prey-predator trophic interactions may be at place. Concerning secondary consumers, we found exceptions within the order of Diptera (i.e., Ceratopogonidae and Tanypodinae). The Diptera order appeared during early Triassic period and are characterized by being fluid feeders [59]. Some primitive groups within the order are primary consumers (e.g., Ptychopteridae) and many species within the large families (e.g., Chironomidae) show aquatic larvae that are primary consumers. We suggest that Ceratopogonidae and Tanypodinae (a subfamily of Chironomidae) show debromination capability, despite being predators, because they evolved from taxa with primary consumer feeding modes possessing the capability [61].

The chordates examined to date in other studies (from fish to cows) all show debromination capability [44], [50], [51], [52], [53], [54], [55]. Chordate early ancestors were primary consumers (herbivores or detritivores) [62]. The debromination capability may have been acquired early in this evolutionary lineage and has not been lost later, even in groups of mostly secondary consumers, either because some selection pressure is maintained (e.g., detoxification of other substances) or because it is related to other functions (e.g., hormonal). However, our results on Br^79^:Br^81^ ratio introduce a new point of view in the consideration of the low BDE-209 levels found in fish as it is the limited transport from the gut to the inner body or, alternatively, fractionation during differential accumulation in certain organs [44].

### BDE-209 Bioaccumulation in Secondary Consumers

The high proportion of BDE-209 in secondary consumers evidence the difficulty for biotransformation in these organisms. However, why do they show such high levels if they are feeding on primary consumers that present very low BDE-209 levels? There are at least two alternative hypotheses: (i) primary consumers are the source and BDE-209 becomes highly biomagnified compared to other congeners, because of its extreme hydrophobicity, or (ii) secondary consumers take up BDE-209 directly from the environment. At first, the former hypothesis appears more likely, since direct uptake from water is doubtful because of the extremely low water-particle partition of BDE-209 [25]. However, it may be argued that even secondary consumers accidentally eat contaminating particles beyond their preferred preys. Bromine isotopic composition of PBDEs sheds light onto the question (Fig. 3). The Br^79^:Br^81^ ratio markedly declines from primary consumers to secondary consumers, which indicates that BDE-209 in secondary consumers is mostly coming from a pool that has been under biotransformation pressure, the BDE-209 molecules with a higher proportion of lighter Br isotopes being more affected. If this were so, the high amount of BDE-209 in secondary consumers would indicate a rather large biomagnification factor for this compound, which agrees with the high hydrophobicity of the molecule. Invertebrate secondary consumers play a key role in freshwater food webs, if they are more affected by the toxic effects of PBDEs, it may eventually result in unbalances and cascading effects in the whole freshwater food web.

### Perspectives

Our study raises many new questions for future research, which cover from PBDE budget in the planet to topics on the molecular mechanisms involved. How relevant is PBDE food web biotransformation in freshwater systems for a PBDE global balance? How extended is the PBDE debromination capability throughout other natural systems (e.g., marine vs. terrestrial)? What are the molecular debromination mechanisms and to what extent may we expect a rapid selective response from organisms not yet showing the capability? Can similar patterns be found for other halogenated contaminants currently assumed as not being biotransformed? Generally, our study shows that the environmental behavior of any new organic chemical is far from being easily predictable. In addition to physico-chemical properties, ecological and evolutionary aspects are involved and have to be considered.

## Supporting Information

Table S1 **PBDE concentrations for each taxon or assemblage (ng g^−1^ dw), mean ± standard deviation.** Number of samples analyzed follow the name. Most samples are composites of a few to many individuals to fulfill analytical requirements. Brown trout (*Salmo trutta*) results have been published previously [34].
(DOC)
