# Supplementary material for: Pollutant Dehalogenation Capability May Depend on the Trophic Evolutionary History of the Organism: PBDEs in Freshwater Food Webs
Source: PLoS One. 2012 Jul 27;7(7):e41829. doi: 10.1371/journal.pone.0041829 (PMC3407054; doi:10.1371/journal.pone.0041829)
Supplement: Table S1 — PBDE concentrations for each taxon or assemblage (ng g−1 dw), mean ± standard deviation. Number of samples analyzed follow the name. Most samples are composites of a few to many individuals to fulfill analytical requirements. Brown trout (Salmo trutta) results have been published previously [34]. (DOC) [file pone.0041829.s001.doc]

**Supplementary Information**

**Table S1:** PBDE concentrations for each taxon or assemblage (ng g^-1^ dw), mean ± standard deviation. Number of samples analyzed follow the name. Most samples are composites of a few to many individuals to fulfill analytical requirements. Brown trout (*Salmo trutta*) results were previously published [29].

| **Sample** | **BDE17** | **BDE28** | **BDE35** | **BDE71** | **BDE47** | **BDE66** | **BDE77** | **BDE100** | **BDE99** | **BDE85** |
| --- | --- | --- | --- | --- | --- | --- | --- | --- | --- | --- |
| *Nostoc* (2) | < 0.0007 | < 0.0004 | 0.0002 ± 0.0002 | < 0.0004 | < 0.0003 | 0.0002 ± 0.0002 | 0.0015 ± 0.0022 | 0.0006 ± 3.1361 | 0.0003 ± 0.0004 | < 0.0004 |
| Epilithon (mainly diatoms and cyanobacteria) (8) | < 0.0007 | < 0.0004 | 0.0033 ± 0.0047 | 0.0042 ± 0.0048 | 0.0471 ± 0.0387 | 0.0009 ± 0.0009 | 0.0328 ± 0.0634 | 0.0098 ± 0.0078 | 0.0344 ± 0.0411 | 6.1889 ± 1.6374 |
| Epipelon (diatoms, cyanobacteria and heterotrophic bacteria) (4) | < 0.0007 | < 0.0004 | 0.0001 ± 0.0002 | 0.0015 ± 0.0012 | 0.0014 ± 0.0013 | 0.0007 ± 0.0010 | 0.0007 ± 0.0008 | 0.0012 ± 0.0016 | 0.0009 ± 0.0007 | < 0.0004 |
| Top sediment (bacterial biofilm with some microalgae) (4) | < 0.0007 | < 0.0004 | 0.0008 ± 0.0017 | 0.0077 ± 0.0070 | 0.0327 ± 0.0326 | 0.0046 ± 0.0092 | 0.0135 ± 0.0155 | 0.0348 ± 0.0253 | 0.0079 ± 0.0065 | 0.0156 ± 0.0145 |
| *Pisidium* (bottom) (3) | < 0.0007 | < 0.0004 | < 0.0004 | 0.0044 ± 0.0077 | 0.0146 ± 0.0097 | < 0.0003 | 0.3572 ± 0.6187 | 0.0481 ± 0.0803 | 0.0043 ± 0.0074 | < 0.0004 |
| *Pisidium* (littoral) (3) | < 0.0007 | < 0.0004 | < 0.0004 | 0.0043 ± 0.0086 | 0.0485 ± 0.0969 | < 0.0003 | < 0.0004 | 0.0159 ± 0.0198 | < 0.0003 | 0.0031 ± 0.0062 |
| *Ancylus fluviatilis* Müller, 1774 (2) | < 0.0007 | < 0.0004 | < 0.0004 | < 0.0004 | 0.0201 ± 0.0184 | < 0.0003 | 0.0039 ± 0.0056 | 0.0211 ± 0.0243 | 0.0035 ± 0.0050 | < 0.0004 |
| *Radix peregra* (Müller, 1774) (13) | < 0.0007 | 0.0055 ± 0.0171 | 0.0072 ± 0.0171 | 0.0263 ± 0.0346 | 0.1382 ± 0.1616 | 0.0092 ± 0.014 | 0.5874 ± 1.0235 | 0.2906 ± 0.3436 | 0.1006 ± 0.1171 | 0.0012 ± 0.0032 |
| *Haliplus,* adult (1) | < 0.0007 | < 0.0004 | < 0.0004 | < 0.0004 | 0.7552 | < 0.0003 | < 0.0004 | 0.0985 | 0.66308 | < 0.0004 |
| Ceratopogonidae (1) | < 0.0007 | < 0.0004 | < 0.0004 | 0.1806 | 0.8332 | < 0.0003 | < 0.0004 | 0.1198 | 0.5335 | < 0.0004 |
| Tanypodinae (4) | < 0.0007 | 0.2555 ± 0.5024 | 0.0062 ± 0.0123 | 0.0445 ± 0.0714 | 1.0280 ± 1.8149 | 0.0096 ± 0.0192 | 0.1877 ± 0.3391 | 0.1954 ± 0.3163 | 0.4748 ± 0.8310 | 0.3753 ± 0.7506 |
| Chironomidae (other than Tanypodinae) (bottom) (3) | < 0.0007 | < 0.0004 | < 0.0004 | 0.0143 ± 0.0248 | 1.0462 ± 1.4952 | < 0.0003 | 6.4361 ± 11.1476 | 0.4414 ± 0.5149 | 0.2092 ± 0.1863 | < 0.0004 |
| Chironomidae (other than Tanypodinae) (littoral) (4) | < 0.0007 | 0.01701 ± 0.0216 | 0.0273 ± 0.0546 | 0.0599 ± 0.0693 | 0.4929 ± 0.4108 | 0.1006 ± 0.1514 | 0.2239 ± 0.2103 | 0.0850 ± 0.1081 | 0.2573 ± 0.2786 | < 0.0004 |
| Planktonic crustaceans (Daphnia longispina ( Müller, 1785), Eudiaptomus vulgaris (Schmeil, 1896), Cyclops abyssorum Sars 1863) (4) | < 0.0007 | 0.0461 ± 0.0922 | 0.0621 ± 0.0992 | 31.185 ± 54.567 | 1.2967 ± 1.4116 | 2.9444 ± 4.4793 | 0.5240 ± 0.3985 | 45.274 ± 83.4582 | 4.9643 ± 7.8992 | 0.2333 ± 0.4667 |
| Oligochaeta (bottom) (3) | < 0.0007 | < 0.0004 | < 0.0004 | 0.1331 ± 0.2305 | 4.6212 ± 4.7776 | < 0.0003 | 0.3863 ± 0.6692 | 23.4001 ± 35.6526 | 1.4180 ± 0.9036 | < 0.0004 |
| Oligochaeta (littoral) (4) | < 0.0007 | < 0.0004 | 0.8012 ± 0.9330 | 0.0514 ± 0.1029 | 3.9276 ± 3.0812 | < 0.0003 | 0.9470 ± 1.1067 | 6.1508 ± 4.9515 | 2.2041 ± 1.4320 | < 0.0004 |
| Hydracarina (1) | < 0.0007 | < 0.0004 | 0.371 | < 0.0004 | 1.8464 | < 0.0003 | 0.0967 | 0.56229 | 1.7262 | < 0.0004 |
| *Aeschna* (1) | < 0.0007 | < 0.0004 | < 0.0004 | 0.0872 | 0.2164 | < 0.0003 | < 0.0004 | 0.0427 | 0.2733 | < 0.0004 |
| Zygoptera (1) | < 0.0007 | < 0.0004 | 0.0258 | 0.20451 | 1.0585 | < 0.0003 | < 0.0004 | 0.0193 | 0.5222 | 0.5602 |
| *Boreonectes*, adult (1) | < 0.0007 | < 0.0004 | < 0.0004 | 0.05589 | 0.0084 | < 0.0003 | < 0.0004 | 0.0135 | 0.0868 | 0.2811 |
| Nematoda | < 0.0007 | < 0.0004 | < 0.0004 | < 0.0004 | 8.2777 | < 0.0003 | < 0.0004 | 3.3257 | 8.912 | 8.726 |
| *Sialis lutaria* (Linnaeus, 1758) (4) | < 0.0007 | < 0.0004 | 0.3746 ± 0.5260 | 0.1090 ± 0.1321 | 0.7991 ± 0.8378 | < 0.0003 | < 0.0004 | 0.6370 ± 0.9681 | 0.5584 ± 0.5979 | 0.0526 ± 0.1052 |
| Limnephilidae (*Annitella, Potamophylax, Limnephilus*) (4) | < 0.0007 | < 0.0004 | 0.0144 ± 0.0279 | 0.0899 ± 0.0939 | 0.9270 ± 0.5576 | < 0.0003 | 0.4667 ± 0.6520 | 0.4317 ± 0.3499 | 0.4822 ± 0.3214 | < 0.0004 |
| *Mystacides azurea* (Linnaeus, 1761) (1) | < 0.0007 | < 0.0004 | < 0.0004 | < 0.0004 | 22.434 | < 0.0003 | < 0.0004 | 4.4014 | 24.6356 | 18.66 |
| *Polycentropus flavomaculatus* (Pictet, 1834) (4) | < 0.0007 | < 0.0004 | 0.2314 ± 0.4310 | 0.2264 ± 0.1619 | 1.4576 ± 1.2582 | < 0.0003 | 0.6482 ± 0.9805 | 0.4038 ± 0.1506 | 0.9034 ± 0.8742 | < 0.0004 |
| *Phoxinus* sp*.* | 0.02307 ± 0.06747 | < 0.0004 | 0.0991 ± 0.2293 | 0.0736 ± 0.1888 | 0.6713 ± 1.1631 | < 0.0003 | 0.0683 ± 0.1212 | 0.0922 ± 0.1233 | 0.3295 ± 0.4858 | 0.0226 ± 0.0265 |
| *Salmo trutta* Linnaeus, 1758 | < 0.0007 | 0.8273 ± 0.5054 | < 0.0004 | < 0.0004 | 1.7643 ± 0.4143 | < 0.0003 | < 0.0004 | 0.7992 ± 0.3548 | 0.9283 ± 0.2043 | < 0.0004 |

| **Sample** | **BDE154** | **BDE153** | **BDE138** | **BDE156** | **BDE183** | **BDE190** | **BDE209** |
| --- | --- | --- | --- | --- | --- | --- | --- |
| *Nostoc* (2) | 0.0003 ± 0.0003 | < 0.0004 | < 0.0004 | 0.0014 ± 0.0014 | 0.0033 ± 0.0046 | < 0.0004 | 0.0630 ± 0.0122 |
| Epilithon (mainly diatoms and cyanobacteria) (8) | 0.0037 ± 0.0035 | 0.0007 ± 0.0019 | 0.0015 ± 0.0017 | 0.0004 ± 0.0008 | 0.0288 ± 0.0491 | 0.0118 ± 0.0080 | 0.1976 ± 0.2459 |
| Epipelon (diatoms, cyanobacteria and heterotrophic bacteria) (4) | 0.0005 ± 0.0003 | 0.0008 ± 0.0004 | 0.0005 ± 0.0010 | 0.0002 ± 0.0002 | 0.0012 ± 0.0015 | 0.0013 ± 0.0015 | 0.0175 ± 0.0189 |
| Top sediment (bacterial biofilm with some microalgae) (4) | 0.0066 ± 0.0093 | 0.0062 ± 0.0054 | 0.0256 ± 0.0189 | 0.0183 ± 0.0205 | 0.0574 ± 0.0516 | 0.0588 ± 0.0803 | 0.0531 ± 0.04408 |
| *Pisidium* (bottom) (3) | < 0.0001 | 0.0275 ± 0.0324 | < 0.0004 | < 0.0004 | 0.1760 ± 0.2730 | < 0.0004 | < 0.0008 |
| *Pisidium* (littoral) (3) | < 0.0001 | 0.0226 ± 0.0257 | < 0.0004 | < 0.0004 | 0.0226 ± 0.0453 | 0.0114 ± 0.0227 | < 0.0008 |
| *Ancylus fluviatilis* Müller, 1774 (2) | < 0.0001 | 0.1111 ± 0.1570 | < 0.0004 | < 0.0004 | 0.0217 ± 0.0093 | < 0.0004 | 0.0134 ± 0.0190 |
| *Radix peregra* (Müller, 1774) (13) | 0.0158 ± 0.0223 | 0.3192 ± 0.4393 | 0.0173 ± 0.0416 | 0.0070 ± 0.0169 | 0.0171 ± 0.0335 | 0.0043 ± 0.0126 | 0.0015 ± 0.0044 |
| *Haliplus,* adult (1) | < 0.0001 | 0.4814 | < 0.0004 | < 0.0004 | < 0.0004 | < 0.0004 | < 0.0008 |
| Ceratopogonidae (1) | < 0.0001 | 0.0226 | < 0.0004 | < 0.0004 | < 0.0004 | < 0.0004 | < 0.0008 |
| Tanypodinae (4) | 0.9597 ± 1.8761 | 0.0683 ± 0.0808 | < 0.0004 | < 0.0004 | 0.0451 ± 0.0609 | 0.0096 ± 0.0192 | < 0.0008 |
| Chironomidae (other than Tanypodinae) (bottom) (3) | < 0.0001 | 0.0907 ± 0.1570 | < 0.0004 | < 0.0004 | 0.0742 ± 0.1285 | < 0.0004 | < 0.0008 |
| Chironomidae (other than Tanypodinae) (littoral) (4) | < 0.0001 | 0.1028 ± 0.1263 | 0.0066 ± 0.0131 | < 0.0004 | 0.1232 ± 0.1575 | < 0.0004 | < 0.0008 |
| Planktonic crustaceans (Daphnia longispina ( Müller, 1785), Eudiaptomus vulgaris (Schmeil, 1896), Cyclops abyssorum Sars 1863) (4) | 0.0862 ± 0.1109 | 0.8588 ± 1.1997 | < 0.0004 | < 0.0004 | 0.1575 ± 0.1897 | 0.0037 ± 0.0075 | 0.3846 ± 0.6696 |
| Oligochaeta (bottom) (3) | < 0.0001 | 4.8405 ± 2.4416 | < 0.0004 | < 0.0004 | 0.0204 ± 0.0354 | 0.0489 ± 0.0846 | < 0.0008 |
| Oligochaeta (littoral) (4) | 0.0327 ± 0.0655 | 8.6348 ± 10.598 | < 0.0004 | < 0.0004 | 0.4462 ± 0.2969 | 0.0088 ± 0.0176 | < 0.0008 |
| Hydracarina (1) | 2.9111 | 1.0014 | 2.71301 | 2.144 | 1.4317 | 2.2238 | 7.3523 |
| *Aeschna* (1) | 0.3722 | 0.19812 | 0.2564 | 0.1529 | 0.2044 | 0.3944 | 0.5732 |
| Zygoptera (1) | 1.1952 | 0.4092 | 0.56 | 0.525 | 0.5167 | 0.4067 | 2.0264 |
| *Boreonectes*, adult (1) | 0.1375 | 0.1421 | 0.2155 | 0.2218 | 0.1487 | < 0.0004 | 0.839 |
| Nematoda (1) | 12.1233 | 4.9043 | 9.069 | 7.2204 | 5.6215 | < 0.0004 | 29.8212 |
| *Sialis lutaria* (Linnaeus, 1758) (4) | 0.9459 ± 1.0920 | 0.8465 ± 1.1228 | 0.1728 ± 0.2407 | 0.34540 ± 0.2971 | 0.4490 ± 0.5841 | 0.3650 ± 0.3795 | 1.2766 ± 1.1216 |
| Limnephilidae (*Annitella, Potamophylax, Limnephilus*) (4) | 0.4566 ± 0.6609 | 0.4573 ± 0.2946 | 0.3926 ± 0.5451 | 0.2586 ± 0.3286 | 0.1825 ± 0.2353 | 0.3232 ± 0.6182 | 0.6620 ± 1.2982 |
| *Mystacides azurea* (Linnaeus, 1761) (1) | 12.5965 | 15.0473 | < 0.0004 | 10.961 | 14.717 | 25.8541 | 35.194 |
| *Polycentropus flavomaculatus* (Pictet, 1834) (4) | 0.6011 ± 0.7272 | 0.8657 ± 1.0794 | 0.0715 ± 0.1430 | 0.2230 ± 0.3284 | 2.0482 ± 3.3936 | 0.3712 ± 0.3986 | 0.8896 ± 1.3005 |
| *Phoxinus* sp*.* | 0.0984 ± 0.1108 | 0.0900 ± 0.0815 | 0.0110 ± 0.0163 | 0.0069 ± 0.0080 | < 0.0004 | 0.0196 ± 0.0390 | 0.0094 ± 0.0188 |
| *Salmo trutta* Linnaeus, 1758 | 0.6280 ± 0.3376 | 0.3149 ± 0.1894 | < 0.0004 | < 0.0004 | < 0.0004 | < 0.0004 | < 0.0008 |
